# Application of a Metal Cobalt Based on 4,6-Bis(imidazol-1-yl)isophthalicacid Metal-Organic -Framework Materials in Photocatalytic CO_2_ Reduction, Antibacterial, and Dye Adsorption

**DOI:** 10.3390/polym15183848

**Published:** 2023-09-21

**Authors:** Yue Han, Lun Zhao, Hongwei Jing, Guanying Song, Ziyun Wang, Jiayu Li, Yi Yang

**Affiliations:** College of Chemistry, Changchun Normal University, Changchun 130032, China; qx202000033@stu.ccsfu.edu.cn (Y.H.); qx202100047@stu.ccsfu.edu.cn (H.J.); qx202100049@stu.ccsfu.edu.cn (G.S.); wangziyun@ccsfu.edu.cn (Z.W.); lijiayu@stu.ccsfu.edu.cn (J.L.); yangyi007@ccsfu.edu.cn (Y.Y.)

**Keywords:** metal-organic frameworks, photocatalytic CO_2_ reduction, antibacterial, adsorption

## Abstract

In this paper, the reported MOF ([Co(bimip)(H_2_O)_0.5_]·0.5H_2_O) was employed in photocatalytic CO_2_ reduction, antibacterial, and dye adsorption experiments. The photocatalytic activity of the MOF for CO_2_ reduction was systematically investigated. The high average CO generation rate of 3421.59 μmol·g^−1^·h^−1^ after 12 h confirms the efficient photocatalytic CO_2_ reduction ability of the MOF. At the same time, the MOF can completely inhibit the growth of *S. aureus* and *C. albicans* within 24 h when its concentration reaches 400 μg/mL and 500 μg/mL, respectively. The MOF has an adsorption capacity for CR. The adsorption rate was 83.42% at 60 min, and the adsorption capacity of the MOF for CR reached 500.00 mg·g^−1^.

## 1. Introduction

In recent decades, CO_2_ emissions have increased dramatically due to the large-scale burning of fossil fuels in industrial production, vehicles, and home stoves. At the same time, massive deforestation by humans has reduced the absorption of CO_2_ by trees, thereby increasing the greenhouse effect [1,2]. From the perspective of photosynthesis, the use of solar energy to convert CO_2_ to hydrocarbons is a potentially effective way to solve the energy crisis and the greenhouse effect at present [3,4]. Therefore, photoactive semiconductor materials have been widely studied by scholars. TiO_2_ has become the first semiconductor material widely studied by scholars due to its stable properties, non-toxicity, and low cost [5]. In addition, various semiconductor materials such as g-C_3_N_4_ [6], CdS [7], SrTiO_3_ [8], and Fe_3_O_4_ [9], etc. [10]. have also been applied to the research of photocatalytic CO_2_ activity. There are various methods of CO_2_ reduction, including photocatalysis [11], electrocatalysis [12], hydrogenation [13], and chemical fixation [14]. Among the numerous methods, photocatalysis has received extensive attention due to its sustainable approach to utilizing solar energy. Different materials have various defects in the photocatalytic CO_2_ reduction process. In order to overcome these defects, the hybrid photocatalytic system exhibits its uniqueness [15,16]. It is worth noting that the use of ruthenium–polypyridineor is ophenylpyridine complexes as photosensitizers, combined with co-catalysts [17,18,19,20] and the mixed system with H_2_O [21] as the solvent for CO_2_ reduction process, has delivered remarkable enhancement effects.

Metal-organic frameworks (MOFs) are novel crystalline materials that form periodic networks with one-, two-, or three-dimensional structures by self-assembly of metal ions or metal clusters with rigid or flexible organic ligands [22,23]. MOFs have potential applications in many aspects due to their special structure and excellent properties, such as catalysis [24], drug delivery [25], solid antibacterial [26], gas separation and storage [27], luminescence [28], and sensors [29], among other fields [30]. For nearly ten years, MOFs with adjustable and high structure stability have been widely discussed in the field of photocatalysis. Li’s group synthesized an amine-functionalized NH_2_-MIL-125(Ti) that could generate HCOO^−^ by carbon dioxide photoreduction in MeCN/TEOA(5/1) solution under visible light irradiation [31]. Subsequently, Zhang et al. introduced the Co site into the porphyrin unit to produce a new composite material, MOF-525-Co. After visible light irradiation, using TEOA as an electron donor, the MOF-525-Co reduced CO_2_ to produce two products, CO and CH_4_ [32]. The Co(II) complex without noble metals has been applied to photocatalytic CO_2_ reduction because of its high light activity in homogeneous solution [33,34,35]. Wang’s research group reasonably designed and prepared a novel 3D BiFeWOx@In_2_S_3_ (BFW@IS-x) core–shell heterostructure, which was used as an efficient photocatalyst for CO_2_ photoreduction. Under the irradiation of visible light, the light conversion efficiency of CO_2_ to CH_4_ and CO on the BFW@IS-1 heterostructure with the best proportion was 49.9 μmol h^−1^ g^−1^ and 28.9 μmol h^−1^ g^−1^, respectively. The CH_4_ production rate of IS 3.5 times and 6.4 times higher than that of pure IS and BFW, and the CO production rate is 4.9 times higher than that of pure IS. Furthermore, the possible mechanism of CO_2_ reduction on BFW@IS-1 composites was discussed [36].

At the same time, with the continuous development and structural optimization of MOFs, they have potential application value in terms of solid antibacterials [37]. Bacteria are the pathogens of many diseases. They can travel through the digestive tract and respiratory tract and achieve contact in a variety of ways, such as transmission, causing disease in normal people [38,39]. They are highly contagious and can cause great harm to society. The antibacterial mechanism of MOFs is mainly due to precipitation of metal cation [40]; the minority is some mild bacteriostasis of organic ligands. Liu et al. synthesized three silver-based MOFs, for *E. coli* and *S. aureus*, which had good antibacterial activity [41]. Shams et al. took *S. aureus* and *E. coli* as objects and used the agar diffusion method to test the bacteriostatic properties of the Cu/H_3_BTC MOF (abbreviated as H_3_BTC). The results showed that the antibacterial effect of H_3_BTC on *S. aureus* was better than that on *E. coli*. The minimum inhibitory concentration test and growth curve test of H_3_BTC also proved its excellent antibacterial properties [42]. In addition, a variety of MOFs have been synthesized with inhibitory effects on *E. coli* and *S. aureus*. Cu_3_(BTC)_2_(BTC = 1,3,5-benzenetricarboxylic acid), reported by researchers such as Abbasi, is synthesized through the layer-by-layer method, and the metal-organic framework (MOF) nanostructure layer technology is formed on silk fibers to alternately corrode Cu(OAc)_2_·2H_2_O and H_3_BTC solutions under ultrasonic radiation. They further studied the effects of pH, reaction time, ultrasonic irradiation, and continuous impregnation steps on the growth of CuBTC organometallic skeleton nanostructures. The results show that silk fibers containing a CuBTC metal-organic skeleton have high antibacterial activity against Escherichia coli and Staphylococcus aureus [43]. The zinc-based metal organic skeleton (MOF) with hydrazine benzoate linker synthesized by the Restrepo research group was evaluated as an antibacterial material against the Gram-positive bacteria Staphylococcus aureus. The material can inhibit the growth and metabolic activities of bacteria, and its half-maximum effective concentration is about 20 mg L^−1^. The material is dispersed in the culture medium. The antibacterial effect can be attributed to the release of 4-hydrazobenzoate linker, and the contribution of free metal can be ignored. The material shows extraordinary durability and can release ligands continuously within a few days [44].

With the continuous development of modern industrialization, the problem of water pollution is becoming increasingly serious. There is a lot of dye wastewater in the water, and its structure is complex, so it is difficult to degrade naturally. Therefore, it is of great significance to explore how to effectively treat dye wastewater [45]. Zhao’s [46] research group successfully synthesized a metal-organic framework (MOF) by ultrasonic-assisted ball milling. In the absence of organic solvents, the coupling effect of ultrasonic wave and mechanical force play an important role in the synthesis of MOFs. In view of adsorption kinetics and isotherm and thermodynamics, the adsorption of Congo red (CR) was studied. The results show that the pseudo-second-order kinetic model and Freundlich adsorption isotherm are a good match for the adsorption of CR on Ni-MOF/GO. The results of adsorption thermodynamics show that the adsorption process is a spontaneous endothermic process. The adsorption capacity of graphene oxide/metal-organic framework (GO/MOFs) for CR reached 2489 mg/g, which was much higher than that reported before. The results show that the increase in the number of active metal sites can significantly improve the adsorption capacity of dyes, and a suitable drying temperature is beneficial to improving the adsorption capacity of dyes. Ultrasonic-assisted ball milling has good adsorption performance and good synthesis prospects.

In this paper, using the MOF ([Co(bimip)(H_2_O)_0.5_]·0.5H_2_O) as catalyst, [Ru(bpy)_3_]Cl_2_·6H_2_O (abbreviated as Ru, bpy = 2′2-bipyridine) as photosensitizer, Triethanolamine (abbreviated as TEOA) as electron donor, and N,N-Dimethylformamide (abbreviated as DMF) and H_2_O as solvent, a photocatalytic CO_2_ reduction system was successfully constructed. Within 12 h, the average generation rate of CO could reach 3421.59 μmol·g^−1^·h^−1^. At the same time, high concentrations of the MOF solution could inhibit the growth of Staphylococcus aureus and Candida albicans within 24 h. In addition, the MOF had a certain adsorption effect on CR, and its removal rate reached 83.42% within 60min.

## 2. Materials and Methods

### 2.1. Materials and Instrumentation

The reagents used in this paper have reagent grade quality and were used without further purification. The purity of the reagents used in this article was above 99%. The infrared spectroscopy (IR) were collected in the 2.5 to 25 mm range with a Thermo Nicolet Avatar 360 IR (Thermo Nicolet Corporation, Waltham, MA, USA) spectrometer. Powder X-ray diffraction (PXRD) data were collected in a D2 PHASER A26-X1 XRD (AXS Co., Ltd., Brooke, Germany) diffractometer with Cu-Kα radiation (λ = 1.54056). The composition and content of CO_2_ reduction products were recorded on a gas chromatograph (GC-7920, Beijing China Education AuLight Technology (CEAuLight) Co., Ltd., Beijing, China). Bacteria liquid under the condition of 600 nm absorbance value (OD_600_) were collected in the SpectraMax Plus 384 (Molecular Devices, San Jose, CA, USA). UV–Vis adsorption spectra were collected in a Cary 300 spectrophotometer (Varian, Palo Alto, CA, USA).

### 2.2. Synthesis of the MOF ([Co(bimip)(H_2_O)_0.5_]·0.5H_2_O)

According to the MOF synthesis method of Li’s team [47], an MOF with the same structure was synthesized in this paper. A mixture of CoCl_2_ (0.0237 g, 0.1 mmol) and 4,6-bis(imidazol-1-yl)isophthalicacid (abbreviated as bimip) (0.0298 g, 0.1 mmol) were added to 8 mL DMF, 2 mL H_2_O, and 1mL 0.5M HNO_3_, respectively, and then, transferred to the autoclave after the solids completely dissolved. To achieve the rapid heating up to 80 °C at 72 h, 10 °C·min^−1^ cooling to room temperature was to obtain purple crystals. The final yield collected was 54 wt% on bimip ligand. Elemental analysis calculated for C_62_H_54_Co_4_N_18_O_24_ (wt%): C, 44.57; H, 3.26; and N, 15.09 was found to be C, 44.69; H, 3.38; N,15.17. FT-IR (KBr pellets, cm^−1^): 3116 (s), 3408 (s), 1663 (w), 1578 (s), 1552 (s), 1393 (w), 1330 (m), 1230 (m), 1090 (s), 857 (m), 824 (s), 787 (m), 665 (s), 611 (s).

### 2.3. Powder X-ray Diffraction (PXRD)

The MOF ([Co(bimip)(H_2_O)_0.5_]·0.5H_2_O) was synthesized according to the synthetic route in the literature, and its XRD data were mild with the literature, indicating that a high purity MOF was obtained (Figure S1, ESI†).

### 2.4. Optimum Reaction Conditions of Photocatalysis

The performance test of the MOF ([Co(bimip)(H_2_O)_0.5_]·0.5H_2_O) for CO_2_ photoreduction was carried out in a closed reactor. Under the irradiation of an Xe lamp (λ ≥ 420 nm), the photoreduction in CO_2_ was carried out by adding the MOF as a catalyst, Ru as a photosensitizer, TEOA as an electron donor, and DMF/DMA/CH_3_CN and H_2_O as reaction solvents. Before lighting, high-purity CO_2_ was continuously circulated for 15 min to ensure that there were no other impurities in the reactor. Then, the reactor was sealed under 1 MPa CO_2_ partial pressure and illuminated with an Xe Lamp for 12 h (λ ≥ 420 nm). During the irradiation process, samples were automatically injected by gas chromatography (GC-7920, FID) every 3 h for analysis. All experiments were repeated five times and averaged. The yield was calculated by PV = nRT, as the ratio of peak area to standard peak area.

### 2.5. Bacteriostatic Ring Experiment and Growth Curve

Staphylococcus aureus (ATCC 6538) and Candida albicans (ATCC 10231) were selected as the analysis object. After inoculating *S. aureus* and *C. albicans* into a conical flask, we cultured them overnight in a shaking table at 37 ℃ and then diluted the bacterial solution to different concentrations for standby. We smeared a certain amount of *S. aureus* and *C. albicans* on LB solid medium and PDA solid medium, respectively. After that, we evenly punched holes with a punch and added 100 μL of the MOF solutions of different concentrations into the hole. We used blank and bimip as the control. The treated agar plate was incubated in a 37 ℃ incubator for 24 h. The diameter of the bacteriostatic ring was recorded.

The antibacterial effect of the MOF was characterized by a growth curve experiment. An appropriate amount of 1 × 10^7^ CFU/mL *S. aureus* and 1 × 10^8^ CFU/mL of *C. albicans* were added to the same volume of different concentrations of the MOF–LB broth medium and the MOF–PDB medium, respectively, and the solution without the MOF sample was used as a blank. All solutions were cultured in a shaking table at 37 ℃. We sucked a sample of the quantitative bacterial solution every 3 h and measure its absorbance (OD_600_) in a 96-well plate to obtain a growth curve of the bacterial solution.

## 3. Results and Discussion

### 3.1. Photocatalytic CO_2_ Reduction

**Figure 1:**
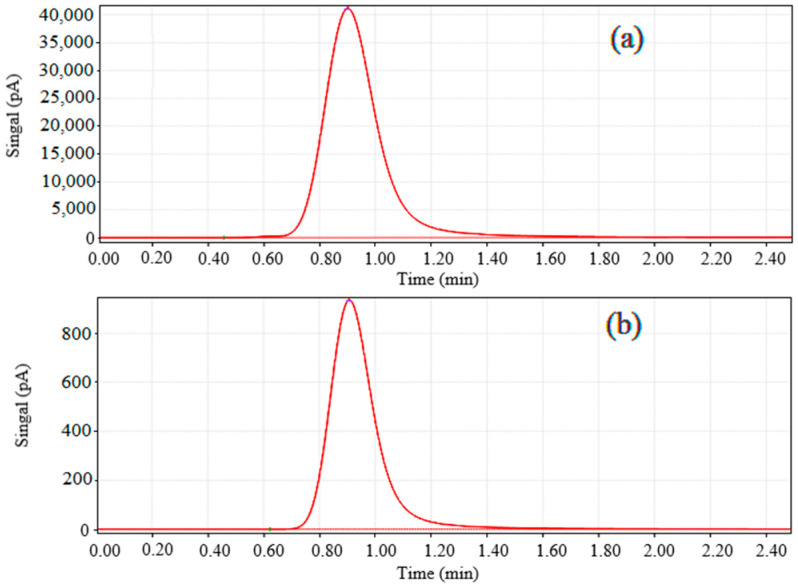
Retention time of (**a**) standard CO and (**b**) reduction product in gas chromatography.

**Figure 2:**
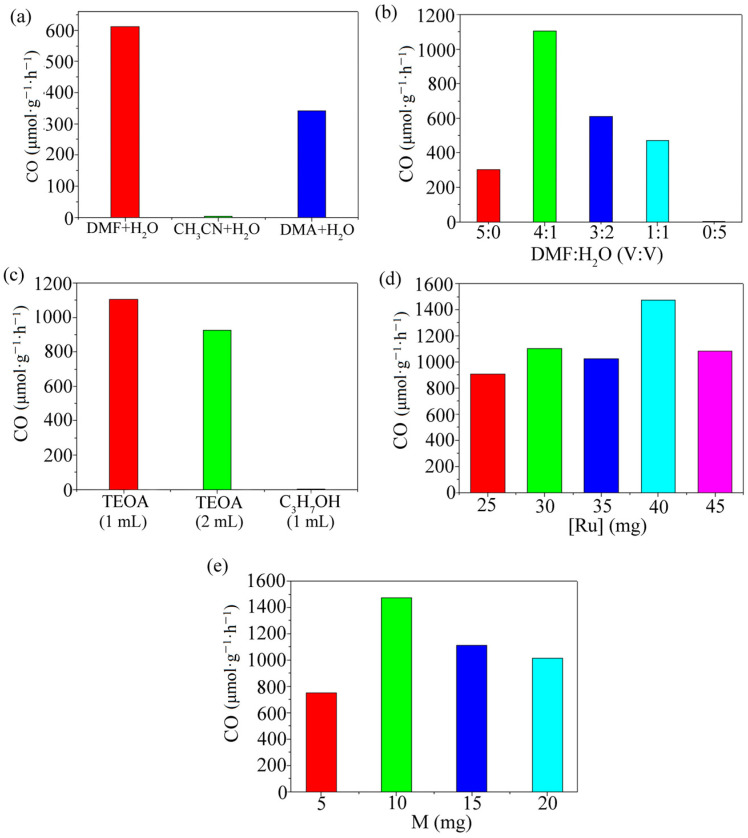
(**a**) CO production rate for different systems; (**b**) C generation rates for different solvent ratios; (**c**) CO generation rates for different types and ratios of electron sacrificial agents; (**d**) CO generation rate at different photosensitizer dosages; (**e**) CO generation rates for different amounts of the MOF.

The reaction product is qualitatively detected as CO by gas chromatography (Figure 1). In order to test the best reaction conditions, DMF, DMA, and CH_3_CN were selected as the reaction solvents by mixing with water. Because the three solvents have good solubility and a high stability for many substances, the effects of different solvents and their proportions on the reaction system were discussed in the experiment. Electronic sacrificial agents play an important role in the photocatalytic system. In this paper, the effects of two sacrificial agents, triethanolamine (TEOA) and isopropanol (C_3_H_7_OH), on the reaction system were investigated. At the same time, the amount of Ru and the MOF was explored to determine the final experimental conditions (Figure 2). The above experiments determined the following optimal reaction conditions for the reaction system: a solution of 10 mg of the MOF, 40 mg Ru, and DMF:TEOA:H_2_O (4:1:1. 6 mL) was added to a 100 mL top-illuminated photocatalytic reactor.

**Figure 3:**
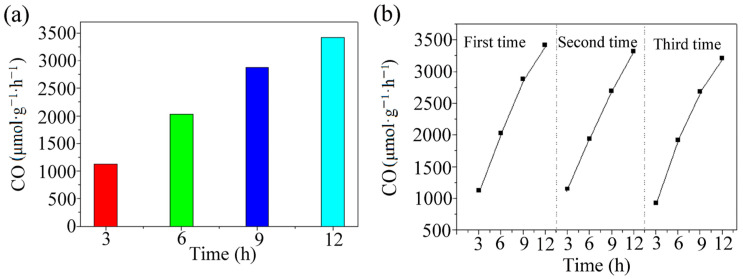
(**a**)Variation in CO formation rate with time in the MOF-catalyzed reaction systems. (**b**) Three-cycle experiments of the photocatalytic reduction system on the MOF.

As the time increased, the amount of CO generated gradually increased, reaching 410.59 μmol within 12 h; the average formation rate was 3421.59 μmol·g^−1^·h^−1^ (Figure 3a). In this paper, the product generation rate of the MOF photocatalytic CO_2_ reduction was compared with the product generation rate under similar conditions (Table S1, ESI†), and the results showed that the MOF photocatalytic CO_2_ reduction performance was excellent.

The light stability of the MOF was investigated through three cycles of photocatalytic CO_2_ reduction. Each cycle was under light for 12 h, and the results are shown in Figure 3b. As shown, the CO production rate decreased negligibly during the cycles. Thus, the MOF had excellent repeatability.

At the same time, in this paper the control experiment was carried out and the results listed in Table 1. In the absence of the MOF, [Ru], and TEOA, the formation rate of CO was very small. In addition, CO was not generated under dark conditions and without CO_2_. This shows that each component in the system is indispensable for photocatalytic CO_2_ reduction, and that the existence of the MOF greatly increases the rate of CO generation, which shows that the MOF plays a vital role in the reaction system.

**Figure 4:**
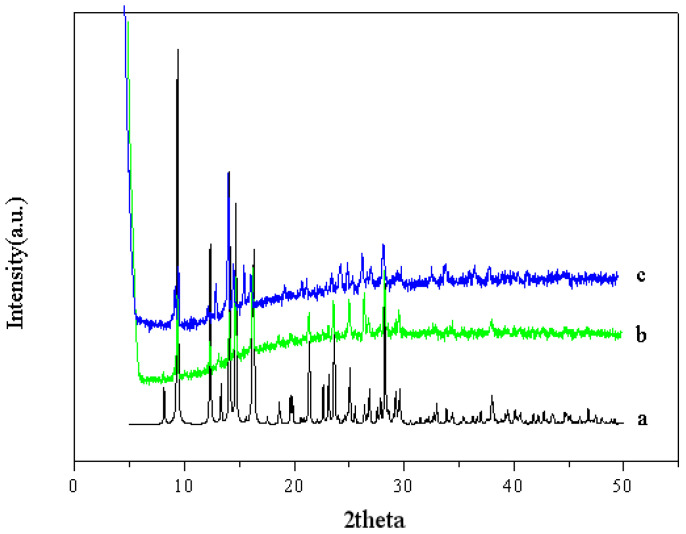
XRD patterns of the MOF (a represents theoretical XRD, b represents experimental XRD, and c represents XRD after three cycles).

In order to prove the stability of the MOF, the recycled MOF was analyzed by powder XRD diffraction. The XRD diffraction patterns show that the structure of the MOF after cycling had almost no change, which indicates that the MOF has good stability (Figure 4).

### 3.2. Antibacterial Properties

Preparing a certain amount of solid culture medium, a proper amount of *S. aureus* diluted to 10^7^ CFU/mL was coated in LB medium; *C. albicans* were coated in PDB medium, evenly. After the bacterial liquid was absorbed, the circular paper soaked with different concentrations (1 mg/mL, 5 mg/mL) of the complex was put into the culture medium. Blank and heterocyclic multidentate carboxylic acid ligands were used as controls. The treated plate was cultured in a constant temperature incubator at 37 °C for 24 h. The diameter of the suppression ring was observed and recorded. The growth of the bacterial liquid in 96-well plate was determined by an enzyme-labeled instrument. Different concentrations of complex solutions were prepared with LB medium, and appropriately diluted Staphylococcus aureus was added. After using PDB medium to prepare complex solutions with different concentrations we added appropriately diluted Candida albicans. The bacterial liquid was put into a shaker at 37 °C, and its absorbance (OD600) at 600 nm wavelength was measured with an enzyme-labeled instrument every 3 h, then, the growth curve of the strain was obtained.

**Figure 5:**
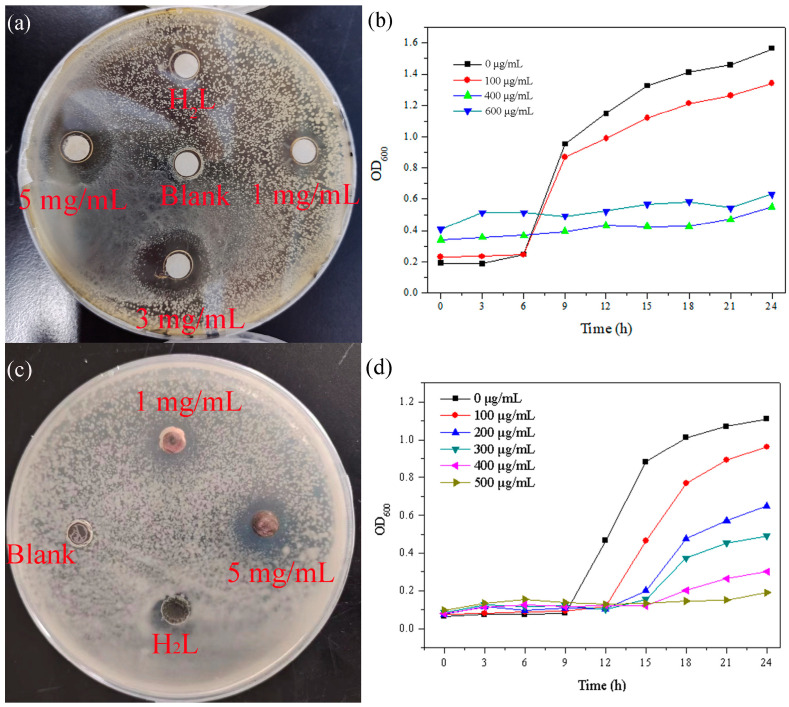
(**a**) Inhibition zone of the MOF against *S. aureus*; (**b**) growth curve of MOF against *S. aureus*; (**c**) inhibition zone of the MOF against *C. albicans*; (**d**) growth curve of the MOF against *C. albicans*.

The experimental results show that metal salts and heterocyclic multidentate carboxylic acid ligands have no bacteriostatic effect on bacterial liquid. The MOF has obvious antibacterial effects on *S. aureus* and *C. albicans* and bimip has no antibacterial effect on the bacterial solution (Figure 5.) The diameter of inhibition zone of 5 mg/mL MOF solution against *S. aureus* and *C. albicans* was 1.2 cm and 1.1 cm, respectively. The growth curve showed that the bacterial liquid in the blank group increased exponentially. For *S. aureus*, low concentration of the MOF could only slightly inhibit its growth; however, when the concentration reached 400 μg/mL, the MOF could completely inhibit the growth of *S. aureus* within 24 h. However, for *C. albicans*, the gradient increase in the MOF concentration gradually enhanced the bacteriostatic effect. When the concentration of the MOF solution was 500 μg/mL, its growth could be completely inhibited within 24 h. With the change in time, the growth of high concentrations of MOF–LB (Figure S2, ESI†) and MOF–PDB (Figure S3, ESI†) bacterial solutions were significantly different from that of blank *S. aureus* and *C. albicans*. The bacterial liquid without the MOF treatment is very turbid, while the bacterial liquid after the MOF treatment is relatively clear, which indicates that the MOF has good antibacterial properties against *S. aureus* and *C. albicans*.

### 3.3. Adsorption Properties of the MOF to Dyes

**Figure 6:**
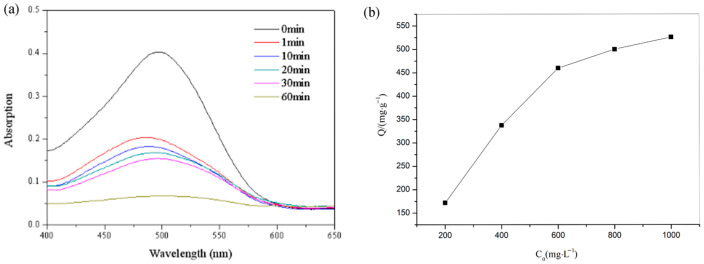
(**a**) UV–Vis spectra of CR with the MOF at different intervals; (**b**) adsorption isotherm curve of the MOF on CR.

With the rapid development of the economy, more and more environmental pollution problems have followed, one of which is dye pollution in water pollution. Dyeing wastewater has a serious impact on organisms, and it must be treated to some extent before it is released into the environment. Dyeing wastewater is non-biodegradable and toxic, and it has been found that long-term contact will cause cancer. Therefore, the treatment of printing and dyeing wastewater has become a global concern. Because the synthesized complex has large porosity, excellent thermal stability, and excellent chemical stability, we tested the adsorption capacity of the MOF for CR. We added 10 mg of the MOF to 50 mL of dye aqueous solution (5 × 10^−5^ M). Then, the ultraviolet-visible spectra were recorded at different time intervals after the mixture reached the adsorption–dissociation equilibrium. The MOF has a good adsorption capacity for CR. The formula r = (C_0_ − Ct)/C_0_ × 100% was used to calculate the dye removal rate, and the adsorption rate was 83.42% at 60 min (Figure 6a). In order to further determine the adsorption characteristics of the complex, the corresponding adsorption isotherms were drawn at room temperature. We added 10 mg of the complex to CR solutions of different concentrations, stirred for 2h to completely adsorb it, and then the ultraviolet spectrum was recorded. According to Q = (C_0_ − Ce) × V/m, the adsorption capacity of the coordination complex for dyes was calculated (where C_0_ is the initial concentration (mg·L^−1^). Ct is the solution concentration at time t (min), (mg·L^−1^), Ce is the equilibrium solution concentration (mg·L^−1^), Q (mg·L^−1^) is the adsorption capacity at different equilibrium solute concentrations, V (L) is the volume of CR, and m (mg) indicates the mass of the complex). The adsorption capacity of the MOF for CR reached 500.00 mg·g^−1^ (Figure 6b).

## 4. Conclusions

In this study, the antibacterial properties, dye adsorption, and photocatalytic CO_2_ reduction properties were studied. The MOF had a certain bacteriostatic effect on *S. aureus* and *C. albicans*, and a high concentration of the MOF could completely inhibit their growth within 24 h. The antibacterial mechanism of the MOF can be attributed to the slow release of metal ions. And the removal rate of CR by the MOF reached 83.42% at 60 min. The high adsorption of CR by the MOF may be due to the adsorption on the surface and pores as well as the Coulomb force between the MOF and CR. Notably, we systematically investigated the photocatalytic CO_2_ reduction performance of the MOF. We successfully constructed a photocatalytic carbon dioxide reduction system using the MOF as a catalyst, Ru as a photosensitizer, TEOA as an electron sacrificial agent, and DMF and water as solvents. Within 12 h, the average generation rate of CO reached 3421.59 μmol·g^−1^·h^−1^. Subsequently, we referred to the relevant literature and think that the reaction mechanism is binuclear metal synergistic catalysis. According to theoretical calculations, one C_O_ (II) acts as the catalytic active center to combine and reduce CO_2_, and the other C_O_(II) acts as the auxiliary catalytic site to promote the decomposition of C-O in the intermediate [O = C-OH] and the departure of -OH, so as to promote the rapid conversion of CO_2_ into CO, thus improving the photocatalytic reaction rate [48]. Because of the porous and large specific surface area of the MOF providing more active sites, it can absorb more CO_2_, which increases the local CO_2_ concentration, thus improving the reaction activity. In summary, the MOF has broad application prospects in the fields of photocatalytic reduction in CO_2_, solid antibacterial, and dye adsorption.

## Figures and Tables

**Table 1:** Control experiments under different conditions.

Entry	[MOF] mg	[{Ru(bpy)_3_}^2+^] mg	TEOA mL	CO μmol·g^−1^·h^−1^
1	10	40	1	2030.26
2	0	40	1	177.52
3	10	0	1	4.13
4	10	40	0	1.37
5 ^a^	10	40	1	0
6 ^b^	10	40	1	0

Reaction conditions in CO_2_-saturated DMF:H_2_O (5 mL, V:V = 4:1) under 6 h of irradiation. ^a^ In the dark. ^b^ Under Ar (without CO_2_).

## Data Availability

Supplementary data associated with this article can be found, in the online version, at https://www.mdpi.com/article/10.3390/polym15183848/s1.

## Supplementary Materials

The following supporting information can be downloaded at: https://www.mdpi.com/article/10.3390/polym15183848/s1, Figure S1: XRD patterns of the MOF (the left is the MOF XRD in the literature; the right is the MOF XRD synthesized in this paper); Figure S2: Changes of MOF solution with different concentration on S.aureus in 24 h; Figure S3: Changes of MOF solution with different concentration on *C. albicans* in 24 h; Table S1: Comparison of production rate of photocatalytic CO_2_ reduction products under similar conditions.

## Sample Availability

Samples of the compounds are available from the authors.
