# Supplementary material for: Application of a Metal Cobalt Based on 4,6-Bis(imidazol-1-yl)isophthalicacid Metal-Organic -Framework Materials in Photocatalytic CO2 Reduction, Antibacterial, and Dye Adsorption
Source: Polymers (Basel). 2023 Sep 21;15(18):3848. doi: 10.3390/polym15183848 (PMC10537682; doi:10.3390/polym15183848)
Supplement: Supplementary file 1 [file polymers-15-03848-s001.zip › polymers-2555981-supplementary.pdf]

Application of a metal cobalt based on 4,6-bis(imidazol-1-yl)isophthalicacid metal-organic framework materials in photocatalytic CO_2 reduction, antibacterial and dye adsorption

Yue Han, Lun Zhao*, ZiyunWang, JiayuLi, Hongwei Jing, Guanying Song, Yi Yang

College of Chemistry, Changchun Normal University, Changchun, 130032, Jilin, P. R. China;

*Correspondence: E-mail: zhaolun@ccsfu.edu.cn; Tel.: +86-431-86168903(L Zhao)

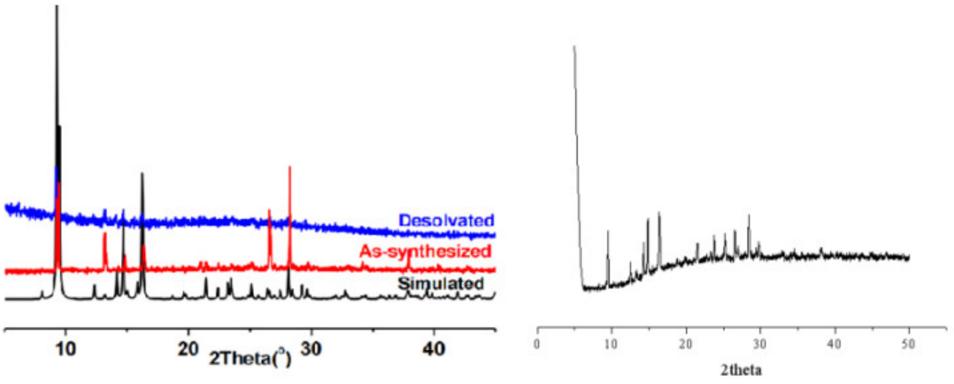

Figure S1. XRD patterns of the MOF (the left is the MOF XRD in the literature; the right is the MOF XRD synthesized in this paper)

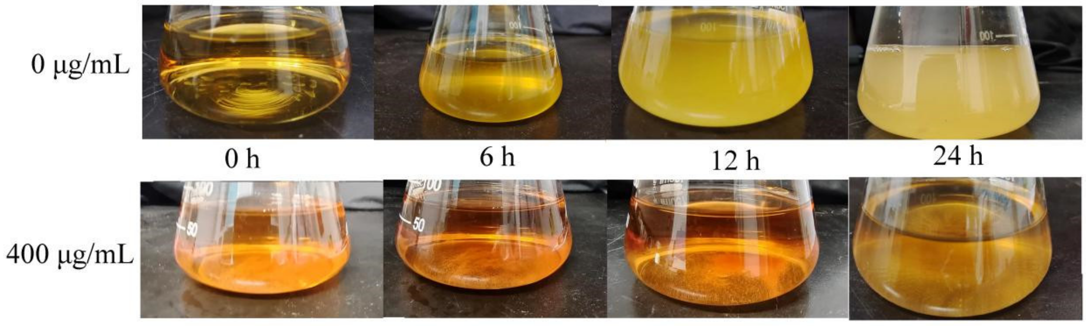

Figure S2. Changes of MOF solution with different concentration on S.aureus in 24 h.

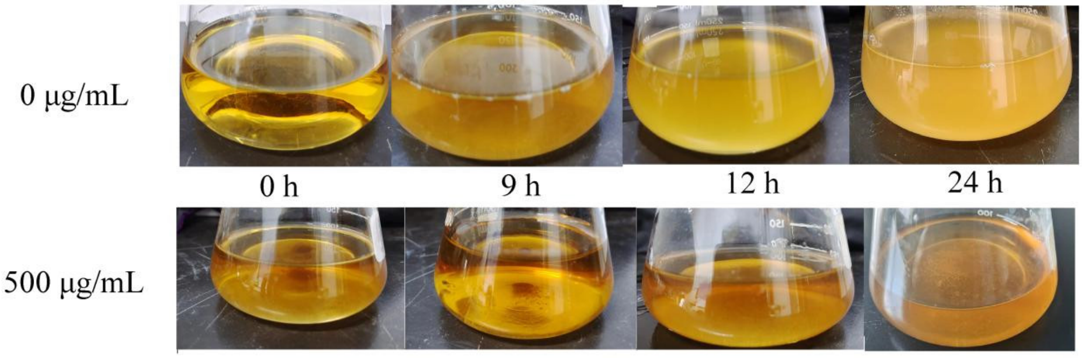

Figure S3. Changes of MOF solution with different concentration on C. albicans in 24 h.

Table S1. Comparison of production rate of photocatalytic CO_2 reduction products under similar conditions

Catalytic agent	Main products	Productivity	Productivity /(μmol/g)	TON	Ref.
ZIF-67@a-TiO_2	CO	43.8 μmol	43800	9.73	Multi-functional amorphous TiO_2 layer on ZIF-67 for enhanced CO_2 photoreduction performances under visible light. Journal of CO_2 Utilization. **2019**, 34, 411-421.
ZIF-67_3	CO	3.89 μmol·mg^{-1}·h^{-1}	15560	3.5	Metal-organic framework (ZIF-67) as efficient cocatalyst for photocatalytic reduction of CO_2: the role of morphology effect. J. Mater. Chem. A, **2018**, 6, 4768-4775.
Ag@Co-ZIF-9	CO	28.4 μmol	28400	-	Photo-reduction of carbon dioxide by ultra-small Ag nanoparticles doped Co-ZIF-9 under visible light. Nanotechnology. **2018**, 29, 284003.
MOF-525-Co	CO	200.6 μmol·g^{-1}·h^{-1}	1203.6	-	Efficient Visible-Light-Driven Carbon Dioxide Reduction by a Single-Atom Implanted Metal–Organic Framework. Angew. Chem. Int. Ed. **2021**, 762, 144101.
TPVT-MOFs@g-C_3N_4	CO	56.4 μmol·g^{-1}·h^{-1}	112.8	-	Metal–organic framework (MOF) composite materials for photocatalytic CO_2 reduction under visible light. Dalton Trans. **2021**, 50, 3186-3192.
$Ni_3(HITP)_2$	CO	3.45×10^4 μmol·g^{-1}·h^{-1}	103500	-	Selective reduction of CO_2 by conductive MOF nanosheets as an effificient co-catalyst under visible light illumination. Applied Catalysis B: Environmental. **2018**, 238, 339-345.
g-C_3N_4-MOLs	CO	464.1 μmol·g^{-1}·h^{-1}	4641	-	Metal-organic layers as a platform for developing single-atom catalysts for photochemical CO_2 reduction. Nano Energy. **2021**, 80, 105542.
[(μ-bdt)$Fe_2(CO)_6$]	CO	-	-	710	Selective photocatalytic reduction of CO_2 to CO mediated by a [FeFe]-hydrogenase model with a 1,2-phenylene S-to-S bridge. Chinese Journal of Catalysis. **2021**, 42, 310-319.

CAT	CO	-	-	140	Selective and Efficient Photocatalytic CO_2 Reduction to CO Using Visible Light and an Iron-Based Homogeneous Catalyst. J. Am. Chem. Soc. **2014**, 136, 16768–16771.
MOF	CO	410.59 μmol	41059.03	68.66	This work
